# Cardio- and Neurometabolic Adipobiology: Consequences and Implications for Therapy

**DOI:** 10.3390/ijms22084137

**Published:** 2021-04-16

**Authors:** Jan Frohlich, George N. Chaldakov, Manlio Vinciguerra

**Affiliations:** 1 International Clinical Research Center, St. Anne’s University Hospital, 656 91 Brno, Czech Republic; jan.frohlich@fnusa.cz; 2 Department of Anatomy and Cell Biology and Research Institute of the Medical University, 9002 Varna, Bulgaria; chaldakov@yahoo.com; 3 Department of Translational Stem Cell Biology, Research Institute of the Medical University, 9002 Varna, Bulgaria

**Keywords:** adipokines, cardiometabolic diseases, Alzheimer’s disease, metabotrophic factors, adiponectin, BDNF, NGF, irisin, Klotho

## Abstract

Studies over the past 30 years have revealed that adipose tissue is the major endocrine and paracrine organ of the human body. Arguably, adiopobiology has taken its reasonable place in studying obesity and related cardiometabolic diseases (CMDs), including Alzheimer’s disease (AD), which is viewed herein as a neurometabolic disorder. The pathogenesis and therapy of these diseases are multiplex at basic, clinical and translational levels. Our present goal is to describe new developments in cardiometabolic and neurometabolic adipobiology. Accordingly, we focus on adipose- and/or skeletal muscle-derived signaling proteins (adipsin, adiponectin, nerve growth factor, brain-derived neuroptrophic factor, neurotrophin-3, irisin, sirtuins, Klotho, neprilysin, follistatin-like protein-1, meteorin-like (metrnl), as well as growth differentiation factor 11) as examples of metabotrophic factors (MTFs) implicated in the pathogenesis and therapy of obesity and related CMDs. We argue that these pathologies are MTF-deficient diseases. In 1993 the “vascular hypothesis of AD” was published and in the present review we propose the “vasculometabolic hypothesis of AD.” We discuss how MTFs could bridge CMDs and neurodegenerative diseases, such as AD. Greater insights on how to manage the MTF network would provide benefits to the quality of human life.

## 1. Introduction

Life at both the local and systemic levels require nutritional, immune, neurotrophic and metabotrophic support. Any dysfunction or deficit in these support systems may lead to serious pathologies, such as cardiometabolic diseases (CMDs), which includes Alzheimer’s disease (AD). Today, CMDs are the number one cause of death globally with phenotypes that encompass atherosclerosis, hypertension, obesity, type 2 diabetes mellitus (T2DM), metabolic syndrome, metabolic-cognitive syndrome and, probably, AD. In fact, growing evidence supports a strong and most likely causal association between CMDs, as well as its risk factors, with incidences of cognitive decline and AD. Individuals with CMDs and subclinical cardiovascular diseases (CVDs) are at higher risk for dementia and AD. Several CMD manifestations, such as obesity, hypertension, high levels of LDL cholesterol, low levels of HDL cholesterol and T2DM are also risk factors for AD [1,2,3]. AD and CMDs are frequently associated. It became clear, through altered insulin/IGF-1 signaling, that impaired systemic insulin signaling and glucose metabolism play a direct role in normal synaptic activity and cognitive function [4]. However, it is necessary to understand the mechanistic effect of cardiometabolic risk factors and circulating mediators on AD in order to better comprehend its pathophysiology. In this short review, we will focus on adipose tissue- or skeletal muscle tissue-secreted metabotrophic factors (MTFs) as potential bridging mechanism between CMDs and AD.

### 1.1. Adipose Tissue

Adipose tissue (AT) is a very plastic and metabolically dynamic organ that is being constantly remodeled to compete with weight gain and weight loss. It is a cellular and extracellular matrix assembly composed of adipocytes, fibroblasts, immune cells and matrix components, and is also rich in sympathetic innervation and stem cells. In the body, there are two major types of AT present. However, clear-cut demarcations do not exist between white adipose tissue (WAT) and brown adipose tissue (BAT), as the infiltration of white adipocytes can be found in BAT (whitening of BAT, a pathogenic phenomenon) and brown adipocytes in WAT (browning of WAT, a sanogenic phenomenon), leading to the formation of brown-in-white AT (brite AT) as well as beige AT [5].

**Figure 1:**
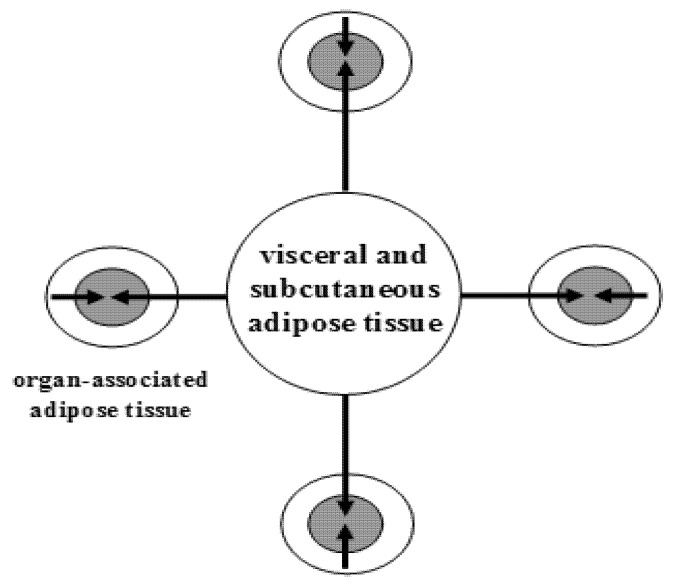
The topography of white adipose tissue. Schematic illustration of a large adipose depot (visceral and subcutaneous adipose tissue) and small adipose depots (organ-associated adipose tissue). Dual action of adipokines, via endocrine pathway (long arrows) and via paracrine pathways (short arrows), on adipose tissue-associated organs is depicted. From: [8] (reproduced with permission).

In humans, WAT is one of the major metabolic and secretory organs that synthesizes and releases a vast number of biologically active compounds that regulate metabolic and cognitive homeostasis (e.g., leptin, adiponectin, visfatin, nerve growth factor (NGF), brain-derived neurotrophic factor (BDNF), neurotrophin-3 (NT-3), nitric oxide (NO), and hydrogen sulfide (H_2_S) [6,7]. WAT is partitioned into two large depots (visceral and subcutaneous), and many small depots associated with various organs, including the heart, blood vessels, major lymph nodes, pancreas, ovaries, bone marrow, eyes, prostate and mammary glands (Figure 1).

### 1.2. Brown, Brite and Beige Adipose Tissue

**Figure 2:**
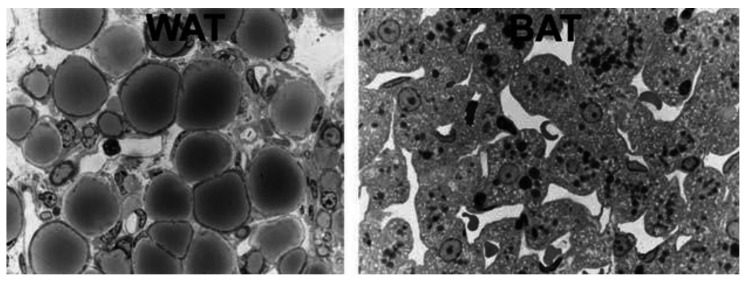
Light microscopy of murine white adipose tissue (WAT), left, and brown adipose tissue (BAT), right. White adipocytes are roundish with unilocular lipid droplets. Brown adipocytes are polyhedral with multilocular lipid droplets and a centrally positioned nucleus. From: [10] (reproduced with permission).

BAT can be visualized using 18F-fluorodeoxyglucose (FDG), an intravenously administered radioactive glucose analog, which is taken up but not metabolized (originally used to delineate metastatic cancers) and viewed using positron emission tomography (PET) scans (Table 1, for light microscopy view, see Figure 2).

### 1.3. Adipobiology: A Research Field Marked by Three Major Paradigm Shifts

One of biggest recent advances in studying obesity and related CMDs is associated with the “rediscovery” of a neglected tissue, the adipose tissue.

The discovery of leptin, an adipose-secreted protein hormone [11], marked a new period of revolutionary science (according to Thomas Kuhn’s 1962 book *The Structure of Scientific Revolutions* [12]) in studying AT. In effect, our understanding of this tissue has, at the epistemological level, undergone three major paradigm shifts in the last 27 years and it has taken the center stage when studying the pathobiology of a vast number of diseases.

**Figure 3:**
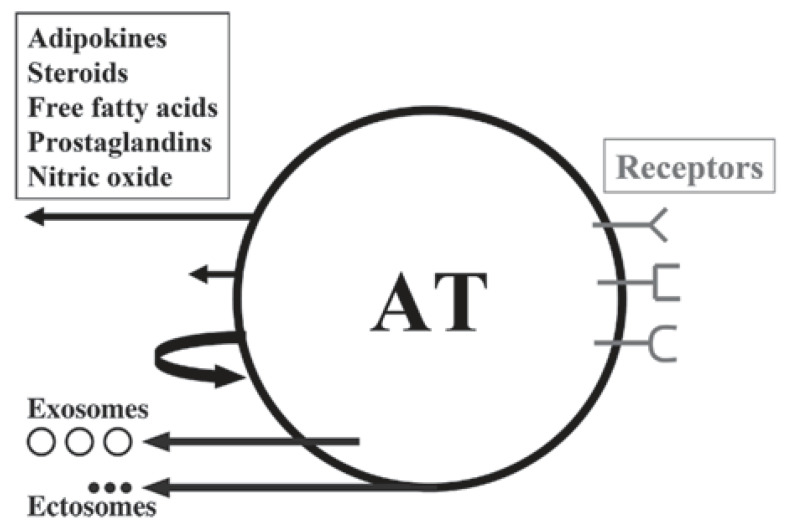
Schematic illustration of white adipose tissue (AT) as a multicrine organ. AT consists of adipocytes, fibroblasts, mast cells, macrophages and other immune cells.The arrows, left from up-to-down, indicate endocrine, paracrine and autocrine pathways; the other two arrows show the extracellular vesicles: exosomes and ectosomes. Depicted on the right are the adipose cell receptors for various ligands. From: [13] (reproduced with permission).

The first paradigm shift revealed that AT, which had for a long time been considered as just an ordinary, inert mass of tissue that only passively stored lipids, was in fact the largest endocrine and paracrine organ of the human body (Table 2, Figure 3).

The second paradigm shift was derived from the studies of Jimmy Bell, head of the Molecular Imaging Group at Hammersmith Hospital in London, UK, who, together with his colleagues, scanned nearly 800 people via magnetic resonance imaging (MRI) to obtain a map of WAT. They demonstrated that as many as 45 percent of women and nearly 60 percent of men who had normal body mass index (BMI) scores (20–25 kg/m^2^) and appeared thin on the outside (TO) actually had excessive levels of internal AT, i.e., they were fat inside (FI). Therefore, they had the TOFI (thin outside, fat inside) phenotype of body fat, which may be considered an “invisible” (hidden) expression of *Homo obesus* (Table 3). Of note, such an adipotopography can be visualized by using current imaging technologies, such as echography, computed tomography, MRI and proton magnetic resonance spectroscopy. These results showed that “being thin does not automatically mean you are not fat,” quoting Dr. Bell. The concept of TOFI holds that small adipose depots, when enlarged and activated (by inflammatory, over-nutritional or other stimuli), may, via a paracrine way, exert disease-promoting actions over AT-associated organ(s) (see Figure 1). Thus, the traditional diagnostic significance of BMI, as well as other anthropometric criteria (waist and hip circumference alike), should be re-evaluated in obesity and its related CMDs. Importantly, dieting may be enough to keep one being thin outside, whereas physical activity prevents the accumulation of internal fat. Thus, the TOFI phenotype is a Trojan horse, a pathological phenomenon, whereas TOTI (thin outside, thin inside) is a healthy adipose phenotype. Briefly, slim or obese, a person should get their AT mapped to evaluate their health status.

**Figure 4:**
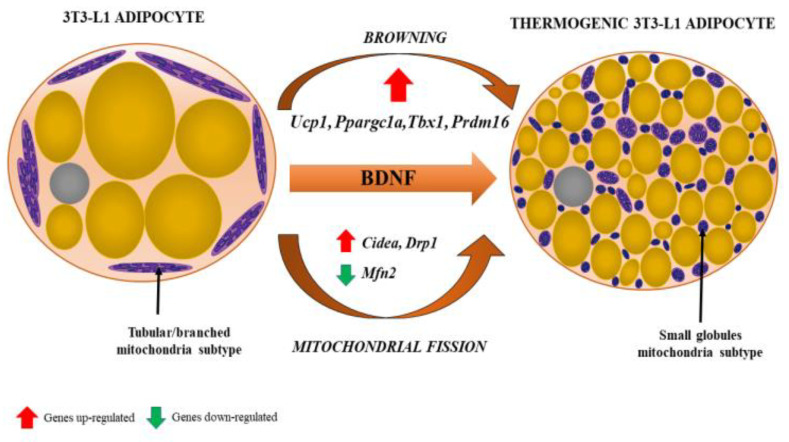
Browning of white adipocyte induced by brain-derived neurotrophic factor (BDNF). The phenotypic change from fat storing to thermogenic adipocytes is recognized by the presence of multilocular lipid droplets and fissed mitochondria that tend to surround lipid droplets, maximizing the efficiency of fatty acid release for thermogenesis. Various genes (their up- and downregulation) involved in the process of browning and mitochondrial fission are indicated by red and green arrows, respectively. From: [18] (reproduced with permission).

The third paradigm shift featured the increasing significance of BAT in both health and disease status. Of note, BAT, with its characteristic small multilocular adipocytes and central nuclei (as opposed to larger unilocular white adipocytes with nuclei at their peripheral rims), as well as granular cytoplasmic appearance on haematoxylin and eosin (H&E) staining, was demonstrated as a component of epicardial AT (EAT) surrounding coronary arteries. Uncoupling protein-1 (UCP-1) is unique to BAT as it uncouples mitochondrial oxidative phosphorylation, thus producing heat instead of ATP by non-shivering thermogenesis, and mRNA**^UCP-1^** is highly expressed in human EAT compared with subcutaneous AT (Figure 4). The amount of BAT identified histologically at autopsy decreases with age and changes its morphology, which is due to the conversion of multilocular brown to unilocular white adipocytes, a process termed adipocyte transdifferentiation [9,14,15,16,17].

## 2. Intermezzo

Giorgio Amendola and Giorgio Napolitano were frequently seen together in Italy in the 1960s and were jokingly called by their friends, respectively, “Giorgio o chiatto” (Giorgio the fat) and “Giorgio o sicco” (Giorgio the slim, though some critics have also referred to him as Re Giorgio, which translates to King George). His “o sicco” status may “explain” why Giorgio Napolitano served as the 11th President of Italy for two terms, from 2006 to 2015. Now, at 96 years of age, he is in a good health. If you buy into the One over Many argument from Plato’s theory of Forms, the power of “o sicco” AT is to be appreciated. At least in Italy.

## 3. Adipokines, Myokines and Metabotrophic Factors

Skeletal muscle tissue may, like AT, function as an endocrine and paracrine organ, secreting multiple signaling proteins called myokines. If secreted by both tissues, they are referred to as adipomyokines (Table 4). The adipomyokines that improve glucose, lipid and energy metabolism were designated metabotrophic factors (from Greek, metabole meaning “a change” and trophe meaning “nutrition,” literally “nutritious for metabolism”) (Table 5).

## 4. Metabotrophic Factors as Therapeutic Targets in Drug Discovery

### 4.1. Trophins Sweet Trophins

In the postgenome era, many “-ome” projects have emerged, including proteome, interactome, metabolome, adipokinome, exposome, connectome and many more. Perhaps this is what prompted Jeff Lichtman and Joshua Sanes to entitle one of their connectome articles “Ome sweet ome” [25].

Mainstream therapies, such as statins, metformin and the like, can improve some symptoms in CMDs, but do not slow progressive atherosclerotic plaque vulnerability and other manifestations of CMDs. Despite intensive research, potential interventions have not demonstrated consistent metabo-protective properties. Thus, patients with CMDs develop severe metabolic degeneration with the resulting morbidity and mortality rates. How can MTFs be targeted for the purpose of the therapy of CMDs? Possible answers might be via: (i) MTF receptor agonists and (ii) boosting intracellular secretory pathways, thus increasing the circulating and/or local levels of MTFs. We suggest that both of these may represent a novel pharmacotherapeutic approach for CMDs. However, our knowledge of the secretory pathways (synthesis, translocation, folding, targeting, sorting, storage and exocytosis) of MTFs remains limited.

### 4.2. Adipomyokines

As was mentioned above, both AT and skeletal muscle tissue are endocrine organs secreting several important signaling proteins, with myokines and adipokines being involved in endocrine and paracrine communications. If myokines and adipokines are secreted by both tissues, they are dubbed adipomyokines (see Table 4). Adipomyokines may exert metabo-protective effects that signify better health [26,27,28].

### 4.3. Adipsin

Adipsin, also known as complement factor D (CFD), was discovered in 1987 [29]. It was the first identified adipose-secreted hormone, a member of the trypsin family of serine proteases, involved in the innate immune defense against infections, as well as insulin secretion and other metabolic functions [30].

### 4.4. Adiponectin

Adiponectin was discovered in 1995 by Yuji Matsuzawa’s research group (reviewed in [31]). It is an anti-diabetic, anti-obesity, anti-inflammatory, anti-atherosclerotic and anti-aging adipokine (in short, a sanogenic “anti-kine”) (see Table 5). Adiponectin has three oligomeric forms, including a trimer (low-molecular weight), hexamer (medium-molecular weight) and high-molecular weight (HMW) form. Among them, HMW adiponectin is the major bioactive form as it displays the greatest insulin sensitizing and anti-inflammatory properties. Blood circulating adiponectin levels in centenarians are associated with an advantageous metabolic phenotype, including increased high-density lipoprotein (HDL) levels, and negatively correlated with C-reactive proteins, which act as an inflammatory biomarker [32].

### 4.5. NGF and BDNF

In 2004, it was reported that NGF is produced by AT [33]; in 2009 it was reported that both NGF and BDNF are produced by AT [34]. Today, it is well known that obesity and related CMDs, including AD, feature reduced circulating and/or local levels of NGF and BDNF. It seems likely hypometabotrophinemia is metabolically harmful, thus staying in the heart of a complex network of factors orchestrating the pathobiology of these disorders [35,36,37,38]. Emerging findings suggest that BDNF has an important and widespread extra-neuronal role in regulating energy homeostasis by controlling feeding and modulating glucose metabolism. BDNF mediates the beneficial effects of energetic challenges, such as vigorous exercise and fasting on cognition, mood and cardiovascular function. By stimulating glucose transport and mitochondrial biogenesis, BDNF supports cellular bioenergetics and protects both metabolism and the brain against injury and disease. Genetic factors, a “couch potato” sedentary lifestyle and chronic stress impair BDNF and NGF signaling, which may contribute to the development of a variety of cardiometabolic and cognitive disorders [37] (see Table 5).

### 4.6. Meteorin-Like (Metrnl)

Meteorin-like (Metrnl; synonyms: cometin, subfatin) is a recently identified adipomyokine that beneficially affects glucose metabolism. It is a blood circulating factor that is induced in muscles after exercise and in AT upon cold exposure. Increasing circulating levels of Metrnl stimulates energy expenditure and improves glucose tolerance and influences expression of genes associated with thermogenesis and anti-inflammatory cytokines. An intraperitoneal injection of recombinant Metrnl improved glucose tolerance in mice with high-fat diet-induced obesity and T2DM. Perhaps Meteorin-like is a promising therapeutic candidate for obesity, T2DM and related CMDs [39,40,41].

### 4.7. Follistatin-Like Protein-1

Follistatin-like protein-1 (FSTL-1) is an adipomyokine that could be a potential regulator of glucose metabolism and insulin resistance in T2DM. FSTL-1 protein expression in the AT of *db*/*db* mice was significantly higher than that of wild-type mice. Circulating FSTL-1 levels in T2DM and obese patients are higher than those in healthy and lean individuals. Physical activity was found to significantly increase the circulating FSTL-1 concentration in young, healthy subjects. Likewise, FSTL-1 protein expression in AT rose dramatically in response to physical activity [42].

### 4.8. Irisin

Irisin (named after the Greek mythology goddess Iris, a messenger of the gods) is a newly identified adipomyokine. It is a cleavage protein of fibronectin type III domain 5 (FNDC5) that is converted to irisin after exercise. Decreased levels of irisin were found to be independently associated with endothelial dysfunction in nonhypertensive, nondiabetic obese subjects [43,44].

### 4.9. Neprilysin

Amyloid precursor protein (APP), amyloid-β (Aβ) peptide and tau hyperphosphorylation are the classical molecular signatures of AD. Of note, it was reported that APP expression was increased in adipocytes in obesity [45]. Among several proteases involved in the proteolysis of Aβ peptide, neprilysin (neutral endopeptidase, NEP), a membrane-associated enzyme, appears to be the most important Aβ-degrading enzyme in the brain. It was reported that human AT-derived stem cells (ADSC) secrete exosomes carrying enzymatically active NEP. When ADSC-derived exosomes were transferred into cultured nerve cells, it resulted in a decrease in both secreted and intracellular Aβ levels [46]. These observations positively indicate the therapeutic relevance of such extracellular vesicles for AD. In sum, (i) both brain tissue and AT have elevated APP levels in obese patients, (ii) there is extraneuronal production of both APPs and Aβ peptides, including in AT, and (iii) the administration of streptozotocin, a well-known experimental model for type 1 diabetes, induces brain insulin resistance and cognitive alterations resembling the status of AD patients [47].

### 4.10. Sirtuins

Sirtuins (SIRT) are proteins encoded by the gene Sir (silent information regulator). The sirtuin family consists of seven members in mammals (SIRT 1–7) [48]. They share nicotinamide adenine dinucleotide (NAD) dependency for their deacetylase activity. Nicotinamide adenine dinucleotide is a coenzyme that consists of adenine and nicotinamide, found in all living cells. NAD exists in two forms: NAD+ and NADH. Nicotinamide phosphoribosyl-transferase (NAMPT) is a NAD biosynthetic enzyme, existing in AT [49,50].

### 4.11. Klotho

Klotho is a transmembrane protein (the enzyme β-glucuronidase) and presents in a secreted, blood circulating form via ectodomain shedding of the membrane-bound form. Soluble Klotho has a variety of salutary effects, including aging suppression. The Klotho proteins, αKlotho and βKlotho, are essential components of endocrine fibroblast growth factor (FGF) receptor complexes, as they are required for the high-affinity binding of FGF19, FGF21 and FGF23 to their cognate FGF receptors (FGFRs). Collectively, these proteins form a unique endocrine network that governs multiple metabolic and cognitive processes in mammals. FGF19 is a satiety hormone that is secreted from the intestine upon ingestion of food and binds the βKlotho-FGFR4 complex in hepatocytes to promote metabolic responses to feeding. By contrast, under fasting conditions, the liver secretes the starvation hormone FGF21, which induces metabolic responses to fasting and stress responses through the activation of the hypothalamus-pituitary-adrenal axis and the sympathetic nervous system following binding to the βKlotho-FGFR complex in adipocytes and the suprachiasmatic nucleus, respectively. Growing evidence suggests that the FGF-Klotho endocrine network also has a crucial role in the pathobiology of aging-related disorders, including CMDs and cognitive deficits. Therefore, targeting the FGF-Klotho endocrine network might have therapeutic benefits in with regards to CMDs and AD [51,52,53,54,55].

### 4.12. Growth Differentiation Factor 11

GDF11 is a member of the TGF-β superfamily of proteins and plays irreplaceable pleiotropic roles in mammalian fetal development [56]. In 2013, GDF11 had been rediscovered and marked as a blood circulating “rejuvenating” cytokine that decreased with age and had the ability to reverse age-related phenotypes in old mice during heterochronic parabiosis experiments [57]. Since then, GDF11 has been heralded as a “fountain of youth” with several studies showing that systemic supplementation of GDF11 levels rejuvenates heart and skeletal muscle tissue in aged mice, reduces atherosclerotic lesion formation, rescues cognitive and cerebrovascular function and ameliorates Aβ levels in mice used to model AD [58,59,60,61]. However, other high-profile studies have seriously questioned these possible rejuvenation effects in aged mice and they showed that supra-physiological doses of GDF11 can promote severe muscle loss, cachexia, fibrosis and premature death [62,63,64,65,66,67]. Recently, research efforts presented GDF11 as an important circulating MTF that significantly ameliorates high-fat diet-induced obesity, hyperglycemia, insulin resistance and liver steatosis by reducing body weight and improving glucose homeostasis [68,69]. Moreover, GDF11 was reported to trigger a calorie restriction-like phenotype by stimulating adiponectin secretion from WAT by direct action on adipocytes [70].

Even though the systemic effects and underlying mechanisms of GDF11 action in obesity and associated metabolic disorders remain poorly understood, further research efforts are essential for the development of safe GDF11-based therapeutic treatments for obesity and related comorbidities (CMDs), including AD.

### 4.13. Neurotrophin-3

As recently reviewed in [71] (and references therein), in a developing brain, neurotrophin-3 and its TrkC receptor are involved in synaptogenesis, but their levels dramatically decrease with brain maturation. In contrast, NT-3 and its TrkC receptor are widely distributed in peripheral tissues, such as blood vessels, heart tissue, AT, pancreas tissue and skeletal muscle tissue. This ubiquitous pattern of presence suggests that (i) NT-3 may exert metabotrophic effects and (ii) small-molecule NT-3 enhancers may emerge as novel drugs for CMDs.

A list of selected MTFs (adipokines, myokines, adipomyokines, etc.) and their respective roles in response to obesity, weight loss, exercise and other serious health factors are presented in the Table 6.

**Figure 5:**
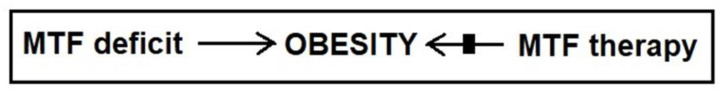
Schematic illustration depicting possible use of MTFs in the therapy of obesity that is currently viewed as an MTF-deficient disease.

**Figure 6:**
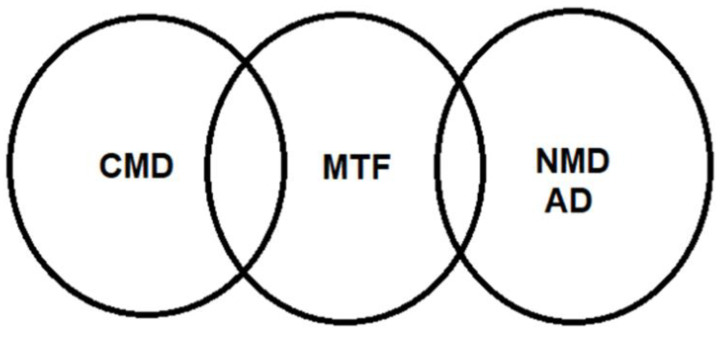
Metabotrophic factors on the crossroad of cardiometabolic diseases (CMDs) and neurometabolic diseases (NMDs), particularly Alzheimer’s disease (AD). Credit to Nikifor N. Chaldakov.

Many basic, clinical and translational studies have demonstrated that both circulating and/or tissue levels of MTFs (metabotrophins, metabokines) are mostly reduced in individuals with obesity and related CMDs, including AD. The scheme within the box illustrates the possible involvement of MTFs in the therapy of obesity, viewed as an MTF-deficient disease (Figure 5), as was particularly suggested and explained in the “prelude” Section 4.1. In this context, Figure 6 illustrates our concept of the potential significance of MTFs for the pathobiology of obesity-related CMDs and neurometabolic diseases (NMDs), particularly AD.

## 5. Coda

Further work in this field could address both CMDs and AD as a single entity rather than two different disorders and “kill two birds with one stone” as Motamedi et al. wrote in [105]; also see [1,2,3,4,37,106,107,108,109,110]. As discussed in [111], clinicians would be able to prescribe to vulnerable patients (identified through genetic analysis or clinical risk stratification) with some CMD drugs that may also exert beneficial effects for neurodegenerative diseases [112,113]. Of note, in 1973 the “vascular hypothesis of AD” was published, whereby cerebral blood hypoperfusion subsequently lead to AD [114], and revitalized now by [111]. In the present review, we propose the “vasculometabolic (metabodegenerative) hypothesis of AD.” Proving or disproving of it must come with further study.

## Figures and Tables

**Table 1:** Topography of brown adipose tissue. From: [9] (reproduced with permission).

Visceral Brown Fat
Perivascular: aorta, common carotid artery, brachiocephalic artery, paracardial mediastinal fat, epicardial coronary artery and cardiac veins, internal mammary artery, and intercostal artery and vein
Periviscus: heart, trachea and major bronchi at lung hilum, esophagus, greater omentum, and transverse mesocolon
Around solid organs: thoracic paravertebral, pancreas, kidney, adrenal, liver, and hilum of spleen
**Subcutaneous Brown Fat**
Between anterior neck muscles and supraclavicular fossa
Under the clavicles
In the axilla
Anterior abdominal wall
Inguinal fossa

**Table 2:** The paradigm shifts in adipobiology.

**From**
Adipose tissue is involved in lipid and energy storage in relation to obesity
**To**
Adipose tissue is an endocrine, paracrine, steroidogenic and immune organIt is a source of and target for inflammatory mediatorsIt produces all components of the rennin-angiotensin systemIt is thus involved in numerous diseases beyond obesity

**Table 3:** Adipotopography: localization of adipose tissue in the human body.

Phenotype	Quality	
TOFI	**	Thin Outside, Fat Inside
TOTI	*****	Thin Outside, Thin Inside
FOFI	*	Fat Outside, Fat Inside
FOTI	***	Fat Outside, Thin Inside

A higher number of asterisks means better quality of health.

**Table 4:** A selected list of adipokines and myokines, some being adipomyokines *.

Irisin—A cleavage protein of fibronectin type III domain 5 (FNDC5) *
Brain-derived neurotrophic factor (BDNF) *
Nerve growth factor (NGF)
Sirtuins, Klotho
Fibroblast growth factor 21 (FGF21) *
Adiponectin *
Follistatin-like protein-1 (FSTL-1) *
Meteorin-like (Metrnl) *
Myonectin
Neprilysin

* A total of 119 myokines, 79 adipokines and 22 adipomyokines were identified in mice [19].

**Table 5:** A selective list of the metabotrophic effects of NGF, BDNF and adiponectin (APN) *.

NGF shares homology with proinsulin
NGF and BDNF are produced by pancreatic beta cells and exert insulinotropic effects
NGF and BDNF are trophic factors for pancreatic beta cells
APN is an anti-obesity, anti-diabetogenic, anti-atherogenic adipokine
NGF, BDNF and APN deficiency led to the development of obesity and related cardiometabolic diseases (CMDs)
NGF, BDNF and APN improve cognitive processes
NGF upregulates expression of LDL receptor-related protein
NGF upregulates expression of PPAR-gamma
NGF inhibits glucose-induced downregulation of caveolin-1
NGF improves skin and corneal wound healing
NGF and BDNF improve vascular (atheroma) wound healing
NGF rescues silent myocardial ischemia in diabetes mellitus
NGF improves diabetic erectile dysfunction
Healthy lifestyle increases brain and/or circulating levels of NGF, BDNF and APN
Atherogenic diet decreases brain BDNF levels

* For references, see [20,21,22,23,24]. Effects of other metabotrophic factors (MTFs) are discussed below.

**Table 6:** A list of endogenous MTFs and their abundance and roles in pathophysiological conditions, such as type 2 diabetes mellitus (T2DM), obesity, CMDs and inflammation.

	Expression Levels		Role in				
	Obesity	Exercise	T2DM	Obesity	CMD	Inflammation	Reference
Adipsin	↑↓	≈ or ↓	↓		↑		[26,27,30,72]
Leptin	↑	↓	↓	↓	↓	↑	[73,74]
Adiponectin	↓	↑	↓	↓	↓	↓	[31,32]
NGF/BDNF	↓	↑	↓	↓	↓		[20,21,22,23,24,35,36,37,38]
Irisin	↑	↑	↓	↓	↓	↓	[43,44,75]
Klotho	↓	↑	↓	↓		↓	[51,52,54,55,76]
FGF21	↑	↑	↓	↓			[51,53,54]
GDF11	≈ or ↑↓	≈ or ↑	↓	↓	↓	↓	[58,59,60,61,68,69,70]
Meteorin-like (Metrnl)	↓	↑	↓	↓		↓	[39,40,41]
FSTL-1	↑	↑	↑		↓	↑	[42,77,78]
Visfatin	↑	↑	↓	↑↓		↑	[79,80]
Humanin	↓	↑	↓		↓		[49,50,81,82]
Omentin	↓	↑	↓	↓	↓	↓	[83,84]
Angiopoietin-like protein 4	↑	↑	↓		↑		[85,86,87]
Aquaporin-7 *	↑	↑	↓	↓	↓		[88,89,90]
Incretins (GLP-1 and GIP)	≈ or↓	↑	↓	↓	↓		[91,92,93]
Kisspeptin-1	↓	↓	↓		↑		[94,95]
Progranulin	↑	≈	↑	↑	↓	↑	[96,97,98,99]
Kallistatin	↓				↓	↓	[100]
Neprilysin	↑		↓		↓	↓	[45,47,101,102]
Myonectin	↓	↑	↓		↓	↓	[103]

Symbols: ≈ unchanged, ↓ decrease/amelioration, ↑ increase/exacerbation, ↑↓ inconclusive. * [17,26,104].

## Data Availability

Not applicable.
